# *Hoxa2* Inhibits Bone Morphogenetic Protein Signaling during Osteogenic Differentiation of the Palatal Mesenchyme

**DOI:** 10.3389/fphys.2017.00929

**Published:** 2017-11-14

**Authors:** Paul P. R. Iyyanar, Adil J. Nazarali

**Affiliations:** Laboratory of Molecular Cell Biology, College of Pharmacy and Nutrition and Neuroscience Research Cluster, University of Saskatchewan, Saskatoon, SK, Canada

**Keywords:** Hoxa2, cleft palate, bone morphogenetic protein (BMP), osteoblast, osteoprogenitor, proliferation, RUNX2

## Abstract

Cleft palate is one of the most common congenital birth defects worldwide. The homeobox (*Hox*) family of genes are key regulators of embryogenesis, with *Hoxa2* having a direct role in secondary palate development. *Hoxa2*^−/−^ mice exhibit cleft palate; however, the cellular and molecular mechanisms leading to cleft palate in *Hoxa2*^−/−^ mice is largely unknown. Addressing this issue, we found that *Hoxa2* regulates spatial and temporal programs of osteogenic differentiation in the developing palate by inhibiting bone morphogenetic protein (BMP) signaling dependent osteoblast markers. Expression of osteoblast markers, including *Runx2, Sp7*, and *AlpI* were increased in *Hoxa2*^−/−^ palatal shelves at embryonic day (E) 13.5 and E15.5. *Hoxa2*^−/−^ mouse embryonic palatal mesenchyme (MEPM) cells exhibited increased bone matrix deposition and mineralization *in vitro*. Moreover, loss of *Hoxa2* resulted in increased osteoprogenitor cell proliferation and osteogenic commitment during early stages of palate development at E13.5. Consistent with upregulation of osteoblast markers, *Hoxa2*^−/−^ palatal shelves displayed higher expression of canonical BMP signaling *in vivo*. Blocking BMP signaling in *Hoxa2*^−/−^ primary MEPM cells using dorsomorphin restored cell proliferation and osteogenic differentiation to wild-type levels. Collectively, these data demonstrate for the first time that *Hoxa2* may regulate palate development by inhibiting osteogenic differentiation of palatal mesenchyme via modulating BMP signaling.

## Introduction

Cleft palate is one of the most common structural birth defects in humans with an incidence of 1 in 700–1,000 live births (Dixon et al., 2011). Studies using mouse model which has a high similarity to human palate development helped to identify several key stages and cellular processes during palate formation (Yu et al., 2017). In mice, secondary palate development begins at embryonic day (E)11.5 and is completed with palatal fusion by E15.5 (Ferguson, 1988). During palate development, the vertical palatal shelves grow downward along the sides of the tongue until E13.5 and then elevate above the tongue at E14. The palatal shelves on either side contact each other forming midline epithelial seam at E14.5, which eventually disintegrates leading to palatal fusion by E15.5 (Kaufman, 1992). Impairment in any of these distinct stages during palatogenesis may result in cleft palate. The palate is comprised of the palatal process of the maxilla and the palatal process of the palatine bone derived from the cranial neural crest cells (CNCC) (Iwata et al., 2010), constituting the anterior and posterior part of the hard palate, respectively (Baek et al., 2011). While the structural changes during palate development are well defined, there is a scarcity of knowledge on the molecular mechanisms governing the patterning of the palate.

In murine models, deletion of about 280 genes are known to cause cleft palate (Funato et al., 2015). Among these genes, mutations of 55 genes are associated with cleft palate in humans (Funato et al., 2015). Mutation in the *Hoxa2* gene is associated with cleft palate in humans (Alasti et al., 2008) and mouse models (Gendron-Maguire et al., 1993; Rijli et al., 1993). In *Hoxa2*^−/−^ mice, the cleft palate phenotype was initially attributed to the physical obstruction of the tongue preventing the palatal shelves to elevate and fuse (Barrow and Capecchi, 1999). However, our group has previously demonstrated that *Hoxa2* is expressed in the palatal shelves (Nazarali et al., 2000) and plays an intrinsic role in palatogenesis (Smith et al., 2009). The palatal shelves from *Hoxa2*^−/−^ mouse embryonic maxilla devoid of tongue grown in rolling bottle cultures failed to fuse (Smith et al., 2009). Hence, tongue musculature may not be the principal reason for the cleft palate phenotype in *Hoxa2*^−/−^ mice. *Hoxa2* appears to be a key regulator of palatogenesis, yet the molecular signaling pathways downstream of *Hoxa2* remain largely unknown.

Bone morphogenetic protein (BMP) signaling plays a critical role in palate development regulating cell proliferation (Zhang et al., 2002). *Bmp4* is upstream of *Bmp2* to induce proliferation in the palatal mesenchyme and is able to reverse the reduced cell proliferation and cleft palate phenotype in the *Msx1*^−/−^ mice (Zhang et al., 2002). Defective cell proliferation observed in *Pax9*^−/−^ embryos is consistent with the reduced *Bmp4* expression in the palatal mesenchyme at E13.5 (Zhou et al., 2013). Similarly, reduced expression of *Bmp2* is associated with reduced cell proliferation in the palatal shelves of *Hand2* hypomorphic mice (*Hand2*^*LoxP*/−^) (Xiong et al., 2009). In addition, growing evidence highlight the importance of osteogenic differentiation in the elevation of palatal shelves and abnormal osteogenic differentiation could lead to cleft palate manifestations (Wu et al., 2008; Fu et al., 2017; Jia et al., 2017a,b). BMP signaling is critical for osteogenic differentiation in the palatal mesenchyme (Wu et al., 2008; Baek et al., 2011; Hill et al., 2014), where it is required for the expression of osteoblast markers such as *Runx2, Sp7*, and *AlpI* (Baek et al., 2011). During craniofacial development, *Hoxa2* restricts the bone mineralization in the calvaria (Dobreva et al., 2006). Moreover, *Hoxa2*^−/−^ mice exhibit ectopic *Runx2*-positive osteogenic center in the second pharyngeal arch that results in duplication of tympanic ring (Kanzler et al., 1998).

In this study, we tested the hypothesis that *Hoxa2* inhibits osteogenic differentiation of the palatal mesenchyme *in vivo* and *in vitro* using *Hoxa2*^−/−^ mice. Our findings reveal that *Hoxa2* plays a critical role in the spatial and temporal regulation of osteogenic differentiation via modulating BMP signaling pathway in the developing palate.

## Materials and methods

### Animals

Wild-type and *Hoxa2*^−/−^ embryos were obtained from timed pregnant *Hoxa2*^+/−^ (heterozygous) mice. Genotype was confirmed using PCR as previously described (Gendron-Maguire et al., 1993). This research was approved by the University of Saskatchewan's Animal Research Ethics Board and adhered to the Canadian Council on Animal Care guidelines for humane animal use.

### Primary mouse embryonic palatal mesenchyme (MEPM) cell culture and osteogenic induction

Primary MEPM cells were isolated from micro-dissected palatal shelves of wild-type and *Hoxa2*^−/−^ mouse embryos at E13.5. The palatal shelves were treated with 0.25% trypsin-EDTA for 15 min, passed through a 70 μm cell strainer and cultured as monolayer cells (Iwata et al., 2012) in DMEM: Ham's F12 (1:1) media containing 10% FBS, 1% antibiotic-antimycotic solution (Sigma). Osteogenic differentiation was carried out as described previously (Kwong et al., 2008) with minor modifications. Briefly, MEPM cells were seeded on 0.1% gelatin or poly-D-lysine coated plates at a cell density of 5 × 10^4^ cells per well in 24 well plates and cultured until they reached confluence. Osteogenic differentiation was induced with differentiation media (DMEM, 10% FBS, 2 mM L-glutamine and 1% antibiotic-antimycotic solution) supplemented with osteogenic inducing agents, including 50 μg/ml L-ascorbic acid 2-phosphate sesquimagnesium salt (Sigma), 10 mM β-glycerophosphate (Sigma), and 100 nM dexamethasone (Sigma). Cells were differentiated for up to 21 days (d) and samples were collected at d8, d15, or d21. To assess the impact of BMP signaling, MEPM cells were treated with dorsomorphin (5 μM) or DMSO and were harvested at d8 for further experiments.

### Alkaline phosphatase I (ALPI) staining

ALPI staining in the palatal shelves *in vivo* was carried out as previously described (Baek et al., 2011). Embryonic mouse heads were fixed overnight in freshly prepared 4% paraformaldehyde at 4°C and rehydrated in 30% sucrose at 4°C. Frozen coronal sections (10 μm) were prepared on slides coated with 0.5% gelatin. The sections were air dried for at least 2 h and then rehydrated with TBS with 0.08% tween-20 two times for 10 min each. Subsequently, the sections were treated with alkaline phosphatase buffer (100 mM NaCl, 100 mM Tris-HCl pH 9.5, 50 mM MgCl_2_, 0.1% Tween-20) for 20 min and stained with alkaline phosphatase buffer containing 4.5 μl/ml of 5-bromo-4-chloro-3-indolyl phosphate (Roche) and 3.5 μl/ml of nitro blue tetrazolium (Roche) for 10 min. The reaction was stopped with PBS containing 20 mM EDTA buffer and counter stained with nuclear fast red. The stained sections were dehydrated in a series of PBS/ethanol, ethanol/xylene and finally mounted in DPX mounting media (Sigma). For osteoblast differentiation in primary MEPM cells, ALPI staining was carried out following the aforementioned protocol after fixing the cells with 4% paraformaldehyde for 15 min.

### Alizarin Red S (ARS) staining and quantification

ARS staining and quantification was carried out as previously described (Gregory et al., 2004) with minor modifications. Briefly, monolayer MEPM cells were fixed with 4% paraformaldehyde for 15 min and stained with 250 μl of 40 mM ARS (Sigma) solution (pH 4.1) at room temperature for 20 min with gentle shaking. Excess dye was aspirated and washed with deionized water before imaging. ARS quantification was carried using an acid extraction method (Gregory et al., 2004). Standard plot of ARS concentration was constructed by serially diluting the 40 mM ARS in the buffer containing 10% (v/v) acetic acid and 10% (v/v) ammonium hydroxide. The absorbance values of the standard concentrations were used to interpolate the concentrations of the test samples.

### Quantitative real-time polymerase chain reaction (qRT-PCR)

Total RNA was isolated from the micro-dissected palatal shelves using RNA mini spin column (Bio-Rad) as per the manufacturer's protocol. First strand complementary DNA synthesis (reverse transcription) was performed in 20 μl reactions with 500 ng of total RNA using High-Capacity complementary DNA Reverse Transcription Kit (Invitrogen). qRT-PCR was carried out as described in our previous study (Thangaraj et al., 2017) using SYBR green assay (Applied Biosystems) in 7300-real time PCR system (Applied Biosystems) with primers listed in Table 1.

**Table 1:** Primer sequences used for the relative quantification of the transcripts by qRT-PCR.

**Transcript**	**Primer sequences**	**Length (bases)**	**Amplicon size (bp)**
Alkaline phosphatase *(AlpI)*	CCTTGACTGTGGTTACTGCT	20	216
	CCTGGTAGTTGTTGTGAGCG	20	
Bone morphogenic protein 2 (*Bmp2*)	CAGTAGTTTCCAGCACCGAA	20	199
	CACTTCCACCACAAACCCAT	20	
Bone morphogenic protein 4 (*Bmp4*)	AGGAAGGAGTAGATGTGAGAG	21	158
	AGGGACGGAGACCAGATAC	19	
Bone carboxy-glutamic acid containing protein (*Bglap*)	GCAGGAGGGCAATAAGGTAG	20	159
	ATGCGTTTGTAGGCGGTC	18	
CyclinD1 (*Ccnd1*)	ACCCTGACACCAATCTCCTC	20	214
	AAGCGGTCCAGGTAGTTCAT	20	
Sp7 transcription factor (*Sp7*)	CACAAAGAAGCCATACGCTG	20	165
	CCAGGAAATGAGTGAGGGAAG	21	
Runt-related transcription factor 2 (*Runx2*)	TGCCTCCGCTGTTATGAAAA	20	187
	CTGTCTGTGCCTTCTTGGTT	20	

### Western blotting

Western bot analyses were carried out as previously described (Brown and Nazarali, 2010). Briefly, palatal tissues were homogenized in RIPA buffer. Total protein content was quantified using the Bradford assay and proteins were separated in 10% SDS-PAGE. Primary antibodies used were: RUNX2 (1:500; Abcam ab102711), SP7 (1:1500; Abcam ab22552), phosphorylated SMAD 1/5/8 (pSMAD 1/5/8) (1:500; Cell signaling 9511S), SMAD 1/5/8 (1:500; Santa Cruz Biotechnology sc-6031-R), and β-ACTIN (1:2,000; Developmental Studies Hybridoma JLA20). Densitometric analyses were carried out using AlphaView software.

### Immunohistochemistry

Embryonic mouse heads were fixed overnight with freshly-prepared 4% paraformaldehyde and rehydrated in 30% sucrose at 4°C. Frozen coronal sections (10 μm) were rehydrated with PBS for 45 min, permeabilized with 0.1% Triton X-100 and blocked with 3% skim milk containing 0.1% Triton X-100 in 1X PBS for 1 h at room temperature. Sections were then incubated overnight with the following primary antibodies: RUNX2 (1:200; Abcam ab23981) or SP7 (1:800; Abcam ab22552) in 1X PBS with 0.1% Triton X-100 at 4°C. Double labeling was carried out by co-incubating: Ki67 (1:100; eBioscience 14569882) and RUNX2 (1:200; Abcam ab23981) overnight at 4°C. Sections were then washed three times and treated with secondary antibodies conjugated with Alexa Fluor® 488 (1:200) or Alexa Fluor® 594 (1:400) in 1X PBS with 0.1% Triton X-100 at room temperature for 1.5 h. Cell counting analyses were carried out manually using ImageJ software platform (NIH).

### Cell proliferation assay

Cell proliferation assay was carried out in MEPM cells using cell-counting kit-8 (Dojindo) as previously described (Iwata et al., 2010). MEPM cells were incubated with CCK-8 reagent for 1 h and the absorbance measured at 450 nm was plotted to calculate the relative cell proliferation rate.

### Statistical analyses

Statistical analyses were carried out using unpaired *t-*test in the case of two groups. One-way ANOVA or two-way ANOVA with Bonferroni multiple comparison test was used for one or two variate analyses, respectively. A *p*-value of <0.05 was considered significant.

## Results

### *Hoxa2^−/−^* mice exhibit increased expression of osteoblast markers during palate development *in vivo*

**Figure 1:**
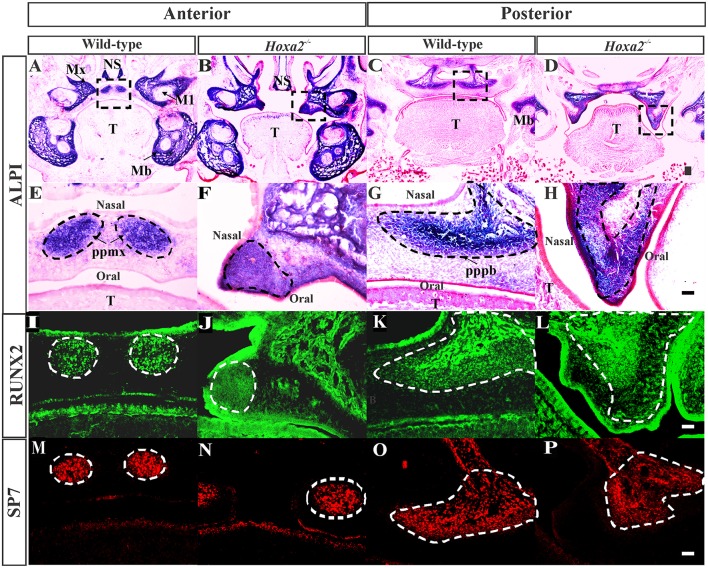
Loss of *Hoxa2* leads to increased osteogenic differentiation of the palatal mesenchyme at E16.5. Position matched coronal sections of wild-type and *Hoxa2*^−/−^ embryos at E16.5 were stained for ALPI **(A–H)**, RUNX2 **(I–L)**, and SP7 **(M–P)**. Sections in the anterior region **(A,B,E,F,I,J,M,N)** were through the middle of the first molar tooth bud to detect osteogenic condensation of the palatal process of the maxilla. Sections in the posterior region **(C,D,G,H,K,L,O,P)** were through the osteogenic centers of the developing palatal process of the palatine bone. **(A–D)** ALPI staining (blue) counterstained with nuclear fast red. Scale bar, 100 μm. Boxed regions in **(A–D)** highlighting the palate are enlarged **(E–H)**. Scale bar, 50 μm. **(E,F)** In the anterior hard palate, ALPI staining in the two condensations of the palatal process of the maxilla (marked in black dotted lines) was evidently increased in the *Hoxa2*^−/−^ embryos **(F)** compared to wild-type **(E)**. **(G,H)** In the posterior hard palate, ALPI stained developing palatal process of the palatine bone (marked in black dotted lines) in the *Hoxa2*^−/−^ embryos **(H)** was increased compared to the wild-type **(G)**, *n* = 5 biological replicates. **(I–P**) Immunohistochemical analyses of RUNX2 (green; **I–L)** and SP7 (red; **M–P)** in wild-type and *Hoxa2*^−/−^ palate at E16.5. RUNX2 was increased in both anterior **(J)** and posterior regions **(L)** of the *Hoxa2* null hard palate, whereas SP7 was increased only in the anterior hard palate **(N)**, *n* = 4 biological replicates. Scale bar, 50 μm. M1, first molar; Mb, mandible; Mx, maxilla; NS, nasal septum; pppb, the palatal process of the palatine bone; ppmx, the palatal process of the maxilla; T, tongue.

To investigate the role of *Hoxa2* in osteogenesis of the palatal mesenchyme, we first examined changes in osteogenic differentiation in the embryonic palatal shelves of *Hoxa2*^−/−^ mice at E16.5, a stage when both the prospective palatal process of the maxilla and the palatal process of the palatine bone evidently ossify (Baek et al., 2011). Staining for ALPI, a marker of osteoblast differentiation showed an expansion in ALPI expression domain in the *Hoxa2*^−/−^ palatal mesenchyme compared to wild-type (Figures 1A–H). At the anterior region of the hard palate, ALPI staining was restricted to the nasal half in two condensations of the prospective palatal process of the maxilla on either side of the degraded midline epithelial seam in wild-type embryos (Figures 1A,E). In contrast, the domain of ALPI positive preosteoblast area was increased and expanded toward the oral side covering oral-nasal axis in *Hoxa2*^−/−^ palatal shelves (Figures 1B,F). In the posterior region of the hard palate, ALPI staining was present in the ossifying centers of the palatal process of the palatine bone in wild-type embryos (Figures 1C,G), whereas there was an expansion in the expression domain of ALPI positive preosteoblasts toward the oral side in *Hoxa2*^−/−^ embryos (Figures 1D,H).

Two well-known regulators of osteogenic differentiation RUNX2 (Komori et al., 1997) and SP7 (previously known as Osterix; Nakashima et al., 2002) have been implicated in the patterning of the palatal bones (Baek et al., 2011). To elucidate the spatial mis-regulation of palatal bone formation in *Hoxa2*^−/−^ mice, expression pattern of these two osteoblast-specific transcription factors were assessed at E16.5. Immunohistochemical analyses revealed that RUNX2 (Figure 1I) and SP7 (Figure 1M) expressions were confined to the condensations of the palatal process of the maxilla at the anterior hard palate in wild-type embryos at E16.5, whereas RUNX2 (Figure 1J) and SP7 (Figure 1N) expression domains were increased in *Hoxa2*^−/−^ embryos. In the posterior region, along the developing palatal process of the palatine bone, the expression of RUNX2 (Figure 1L) was increased in *Hoxa2*^−/−^ embryos compared to wild-type (Figure 1K). In this region, the expression of SP7, a downstream target of RUNX2 and a marker of mature osteoblasts, was not evidently increased in the *Hoxa2*^−/−^ palate (Figure 1P) compared to wild-type (Figure 1O). This suggests that cells toward the oral side of the palatal process of the palatine bone are at immature osteoblast stage and may not have developed bone matrix by E16.5. Collectively, these data indicate that *Hoxa2* could be a potential inhibitor of osteogenic differentiation in the palatal mesenchyme, which may serve to spatially restrict the expression of osteoblast-specific proteins during palate development *in vivo*.

**Figure 2:**
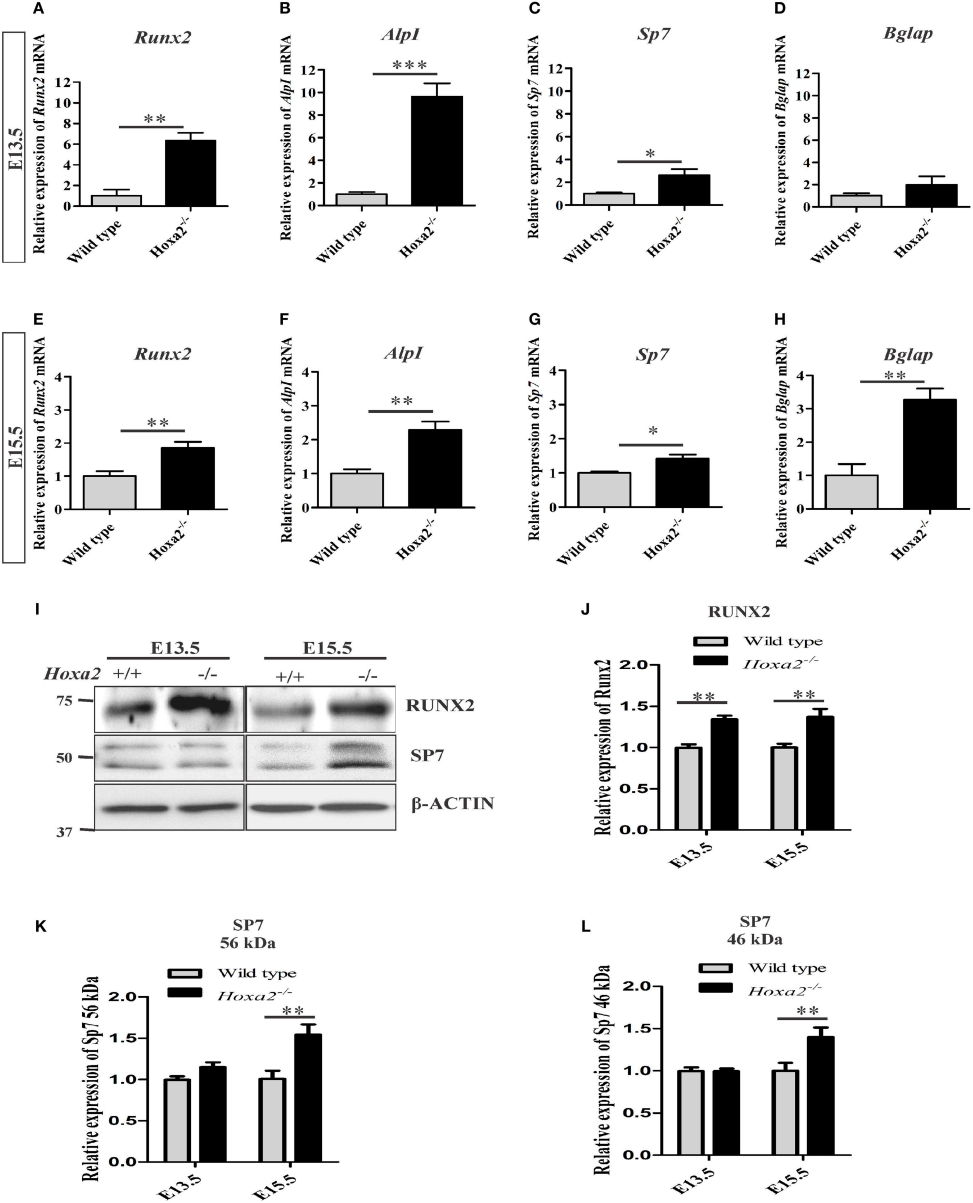
Loss of *Hoxa2* leads to increased expression of osteogenic markers in the developing palate at E13.5 and E15.5. Quantitative real-time PCR (qRT-PCR) analyses indicate that the gene expression profile of osteogenic markers such as *Runx2* **(A,E)**, *AlpI* **(B,F)**, and *Sp7* **(C,G)** were upregulated in the developing *Hoxa2*^−/−^ palatal shelves at E13.5 **(A–C)** and E15.5 **(E–G)**. Gene expression of *Bglap* was upregulated at E15.5 **(H)** but not at E13.5 **(D)**. qRT-PCR data (*n* = 5 biological replicates) were normalized to β*-actin* and represented relative to wild-type (mean ± S.E.M; unpaired *t*-test, ^*^p < 0.05; ^**^p < 0.01; ^***^p < 0.001). Western blot analyses of RUNX2 **(I,J)** and SP7 **(I,K,L)** were carried out using the microdissected palatal shelves from wild-type and *Hoxa2*^−/−^ mice at E13.5 and E15.5. RUNX2 protein expression was upregulated in the *Hoxa2*^−/−^ palate at E13.5 and E15.5 **(I,J)**, whereas SP7 isoforms were upregulated at E15.5 **(I,K,L)**. Densitometric analyses (*n* = 4 biological replicates) were normalized to β-ACTIN and represented relative to wild-type (mean ± S.E.M; unpaired *t*-test, ^**^p < 0.01).

Furthermore, gene expression profiles of osteoblast markers were evaluated during the initiation of ossification of the palatal process of the palatal bone and the palatal process of the maxilla at E13.5 and E15.5, respectively. The loss of *Hoxa2* in the developing palate resulted in an increase in mRNA expression of osteoblast markers such as *Runx2, AlpI* and *Sp7* at both E13.5 (Figures 2A–D) and E15.5 (Figures 2E–H). At E13.5, mRNA expression of *Runx2, AlpI* and *Sp7* were increased to ~6.36-, ~9.65-, and ~2.62-fold, respectively, in *Hoxa2*^−/−^ palate compared to wild-type (Figures 2A–C). At E13.5, mRNA expression of *Bglap* (previously known as *Ocn*) was not significantly altered (Figure 2D). At E15.5, mRNA expression of *Runx2, AlpI, Sp7*, and *Bglap* were upregulated ~1.86-, ~2.29-, ~1.42-, ~3.27-folds, respectively, in *Hoxa2*^−/−^ palate compared to wild-type (Figures 2E–H). Consistent with this, protein expression of RUNX2 was upregulated at E13.5 and E15.5 to ~1.4-fold (Figures 2I,J). SP7 protein expression, both long (Figures 2I,K) and short isoforms (Figures 2I,L) were upregulated at E15.5 to ~1.4-fold. These data reveal that along with regulating the spatial patterning of osteogenic differentiation, *Hoxa2* also regulates the expression of osteogenic markers at the molecular level in the developing palate.

### *Hoxa2* inhibits osteoblast differentiation of mouse embryonic palatal mesenchymal (MEPM) cells *in vitro*

**Figure 3:**
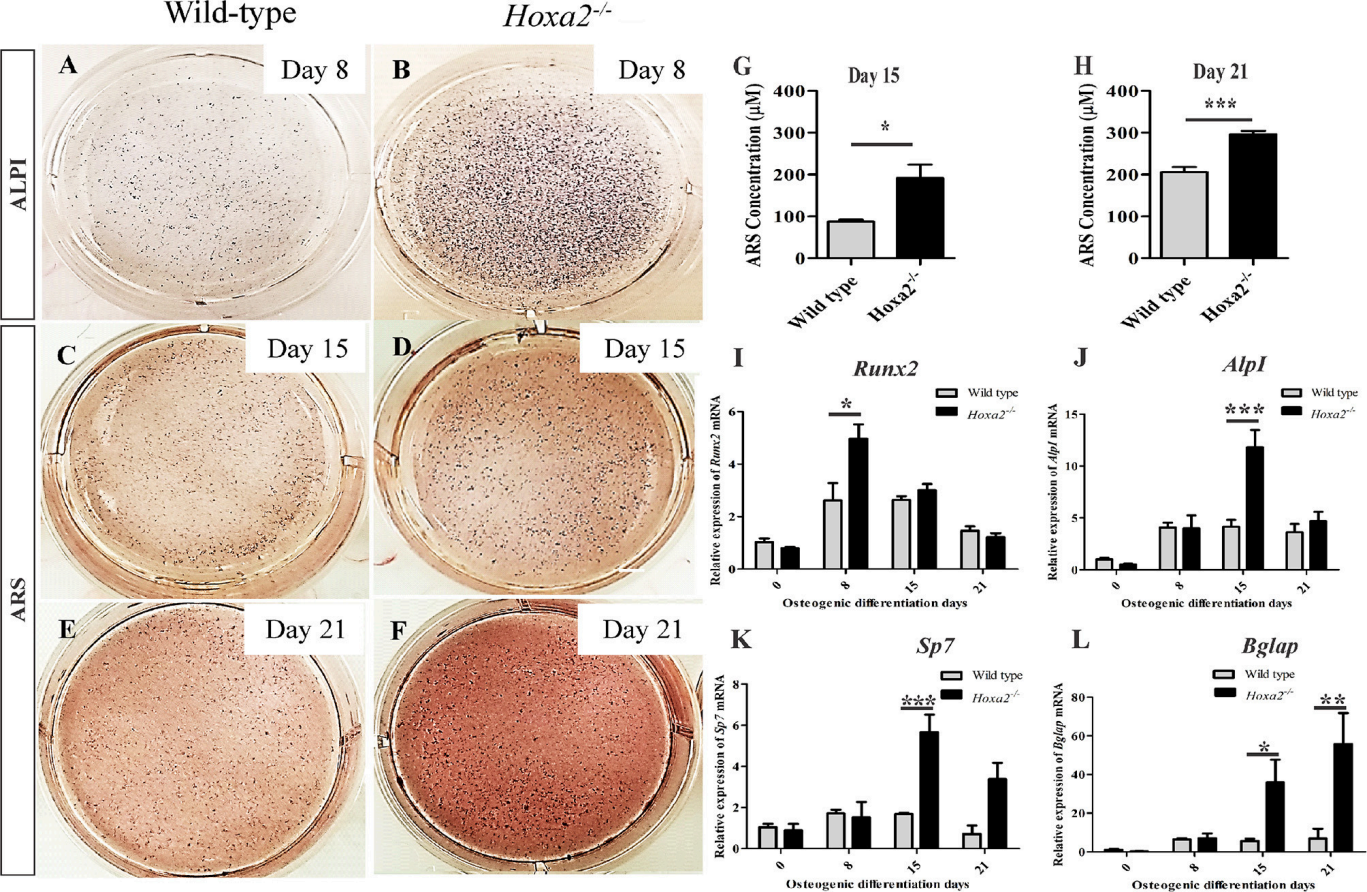
*Hoxa2* inhibits osteoblast differentiation of MEPM cells *in vitro*. Wild-type and *Hoxa2*^−/−^ MEPM cells were subjected to osteogenic differentiation *in vitro* for up to 21 days (d). The differentiated cells were stained for ALPI at d8 **(A,B)**, ARS at d15 **(C,D)**, and d21 **(E,F)**. ARS stained osteocyte matrices from wild-type and *Hoxa2*^−/−^ MEPM cells were extracted and quantified at d15 **(G)** and d21 **(H)**. Experiment was carried out three times and the data shown here are from a representative experiment with *n* = 3 biological replicates; mean ± S.E.M; unpaired *t*-test, ^*^p < 0.05; ^***^p < 0.001). *Hoxa2*^−/−^ MEPM cells displayed increased matrix deposition and mineralization at d15 **(G**) and d21 **(H)**, respectively. **(I–L**) qRT-PCR analyses revealed that gene expression profile of osteogenic markers such as *Runx2* **(I)**, *AlpI* **(J)**, *Sp7* **(K)**, and *Bglap* **(L)** were upregulated in the *Hoxa2*^−/−^ MEPM cells in a stage-specific manner during osteoblast differentiation. Data was normalized to β*-actin* and represented relative to wild-type at d0 (*n* = 3 biological replicates; mean ± S.E.M; two-way ANOVA with Bonferroni post-hoc test, ^*^p < 0.05; ^**^p < 0.01; ^***^p < 0.001).

To evaluate the potential of *Hoxa2* in regulating the temporal pattern of osteogenesis, the primary mesenchyme cells from the wild-type and *Hoxa2*^−/−^ palatal shelves were differentiated *in vitro* for up to 21 days (d). Osteogenesis of mesenchymal cells involves sequential stages of proliferation, osteogenic commitment around day8 (~d8) followed by matrix deposition (~d15) and mineralization (~d21) (Gordon et al., 2010). Differentiating cells were stained for ALPI at d8 and Alizarin Red S (ARS) at d15 and d21. ALPI staining showed an increased osteoblast differentiation at d8 in *Hoxa2*^−/−^ MEPM cells compared to the wild-type MEPM cells (Figures 3A,B). In addition, ARS staining followed by quantification of ARS extracted matrix showed that *Hoxa2*^−/−^ MEPM cells exhibited increased extracellular matrix deposition ~2-fold at d15 (Figures 3C,D,G) and increased mineralization ~1.5-fold at d21 (Figures 3E,F,H) compared to the wild-type MEPM cells.

Next, the gene expression profiles of osteogenic markers were examined in wild-type and *Hoxa2*^−/−^ MEPM cells during osteogenic differentiation *in vitro*. *Runx2* mRNA expression was increased to ~1.9-fold in the *Hoxa2*^−/−^ MEPM cells compared to the wild-type during osteoblast commitment stage at d8 (Figure 3I). *AlpI* and *Sp7* mRNA expression were increased ~2.85 and ~3.37-fold, respectively, during matrix deposition stage at d15 in the *Hoxa2*^−/−^ MEPM cells (Figures 3J,K). *Bglap* mRNA expression was increased ~6.37-fold during matrix deposition at d15 and ~8.09-fold during matrix mineralization at d21 in the *Hoxa2*^−/−^ MEPM cells (Figure 3L). Thus, loss of *Hoxa2* results in upregulation of osteogenic marker expression in a stage-specific manner as early as d8 (osteogenic commitment stage). These data indicate that *Hoxa2* may play a role in early osteoblast differentiation by inhibiting the transcription factors regulating osteogenic fate specification.

### Increased osteoprogenitor proliferation and commitment in the *Hoxa2^−/−^* palatal mesenchyme during early palate development

**Figure 4:**
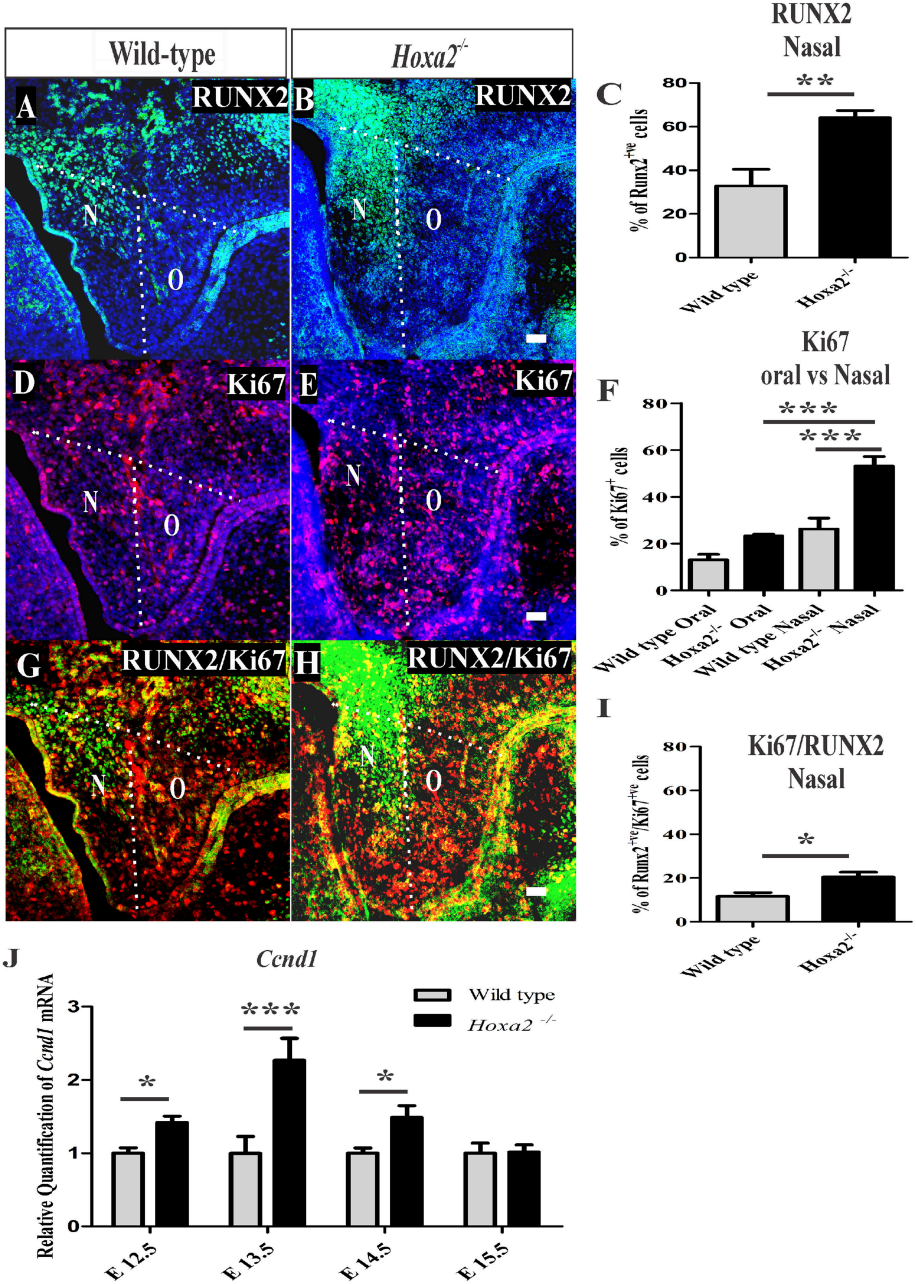
*Hoxa2*^−/−^ palatal shelves exhibit increased osteoprogenitor proliferation and commitment at E13.5. Osteoprogenitor cells in the developing palatal shelves of wild-type and *Hoxa2*^−/−^ embryos were evaluated using RUNX2 immunostaining **(A,B)** and RUNX2-positive cells were counted manually using ImageJ platform **(C)**. Proliferation rate was assessed using Ki67 immunostaining **(D–F)** at E13.5. Scale bar, 50 μm; N, nasal; O, oral. Proliferating osteoprogenitor cells (cellspositive for both RUNX2 and Ki67) (Runx2 /Ki67) **(G,H)** relative to the total number of mesenchymal cells (DAPI-positive) from wild-type and *Hoxa2*^−/−^ palatal shelves were counted in the nasal side **(I)**. *Hoxa2*^−/−^ embryos exhibited increased RUNX2-positive **(C)**, Ki67-positive **(F)** and RUNX2/Ki67-double positive **(I)** cells in the nasal side of the palatal shelves (*n* = 5 biological replicates; mean ± S.E.M; unpaired *t*-test, ^*^p < 0.05; ^**^p < 0.01; ^***^p < 0.001). Expression of cell cycle regulator *Cyclin D1* (*Ccnd1*) mRNA was upregulated in the *Hoxa2*^−/−^ palatal shelves **(J)** from E12.5 to E14.5. qRT-PCR data was normalized to β*-actin* and represented relative to wild-type at respective embryonic stages (*n* = 6 biological replicates; mean ± S.E.M; unpaired *t*-test, ^*^p < 0.05; ^***^p < 0.001).

*Hoxa2* peaks in its expression in the developing palate at E13.5 (Smith et al., 2009), a stage when the mesenchymal cells simultaneously proliferate and commit to form preosteoblasts of the prospective palatal process of the palatine bone. This suggests that the cleft palate phenotype in *Hoxa2*^−/−^ mice, due to the failure of palatal shelves to elevate and reorient horizontally above the tongue after E13.5 (Barrow and Capecchi, 1999), may be a consequence of abnormal cell proliferation (Smith et al., 2013) and osteogenic differentiation (Wu et al., 2008; Fu et al., 2017; Jia et al., 2017a,b). To gain further insight into the role of *Hoxa2* during this early stage of palate development, the rate of mesenchymal cell proliferation and the commitment of mesenchymal cells to osteoprogenitor fate was investigated *in vivo* at E13.5. Immunohistochemical staining of RUNX2 (Figures 4A,B) was used to evaluate osteoprogenitor commitment in the wild-type and *Hoxa2*^−/−^ palatal shelves at E13.5. RUNX2 (Figure 4A) expression in the wild-type was restricted to the bend region in the nasal side of the palatal shelves, whereas the expression domain of RUNX2 (Figure 4B) was increased spatially toward the medial edge of the palate as well as to the oral side of the palate in the *Hoxa2*^−/−^ mutants. This is similar to the aberrant expression patterns of RUNX2 observed at E16.5 in the *Hoxa2*^−/−^ palatal shelves. In addition, the number of RUNX2-positive osteoprogenitor cells on the nasal side of the palatal shelves were significantly higher in the *Hoxa2*^−/−^ mutants (~64%) compared to wild-type (~33%; Figure 4C).

Next, the rate of cell proliferation was assessed at E13.5 using Ki67 immunostaining. The percentage of Ki67-positive cells was significantly increased in the *Hoxa2*^−/−^ palatal mesenchyme (~50%) compared to wild-type (~26%; Figures 4D–F). In the nasal side of the palatal shelves, the percentage of Ki67-positive cells was ~53% in *Hoxa2*^−/−^ embryos compared to ~26% in the wild-type (Figure 4F). Interestingly, the nasal side mesenchyme displayed a higher proliferation rate of ~53% compared to the oral side of ~23% in *Hoxa2*^−/−^ palatal shelves (Figure 4F). In addition, the percentage of proliferating osteoprogenitor cells (RUNX2-positive/Ki67-positive) in the nasal side of the *Hoxa2*^−/−^ palatal shelves was higher (~20%) compared to ~11% in wild-type (Figures 4G–I). Furthermore, mRNA expression of cyclin D1 (*Ccnd1*), a critical G1 phase cell cycle regulator was also upregulated in the *Hoxa2*^−/−^ palatal shelves from E12.5 to E14.5 (Figure 4J). These results indicate that *Hoxa2* plays a critical role by inhibiting osteoprogenitor commitment and osteoprogenitor proliferation prior to the elevation and fusion of the palatal shelves.

### Increased canonical BMP signaling pathway in the *Hoxa2^−/−^* palatal shelves

**Figure 5:**
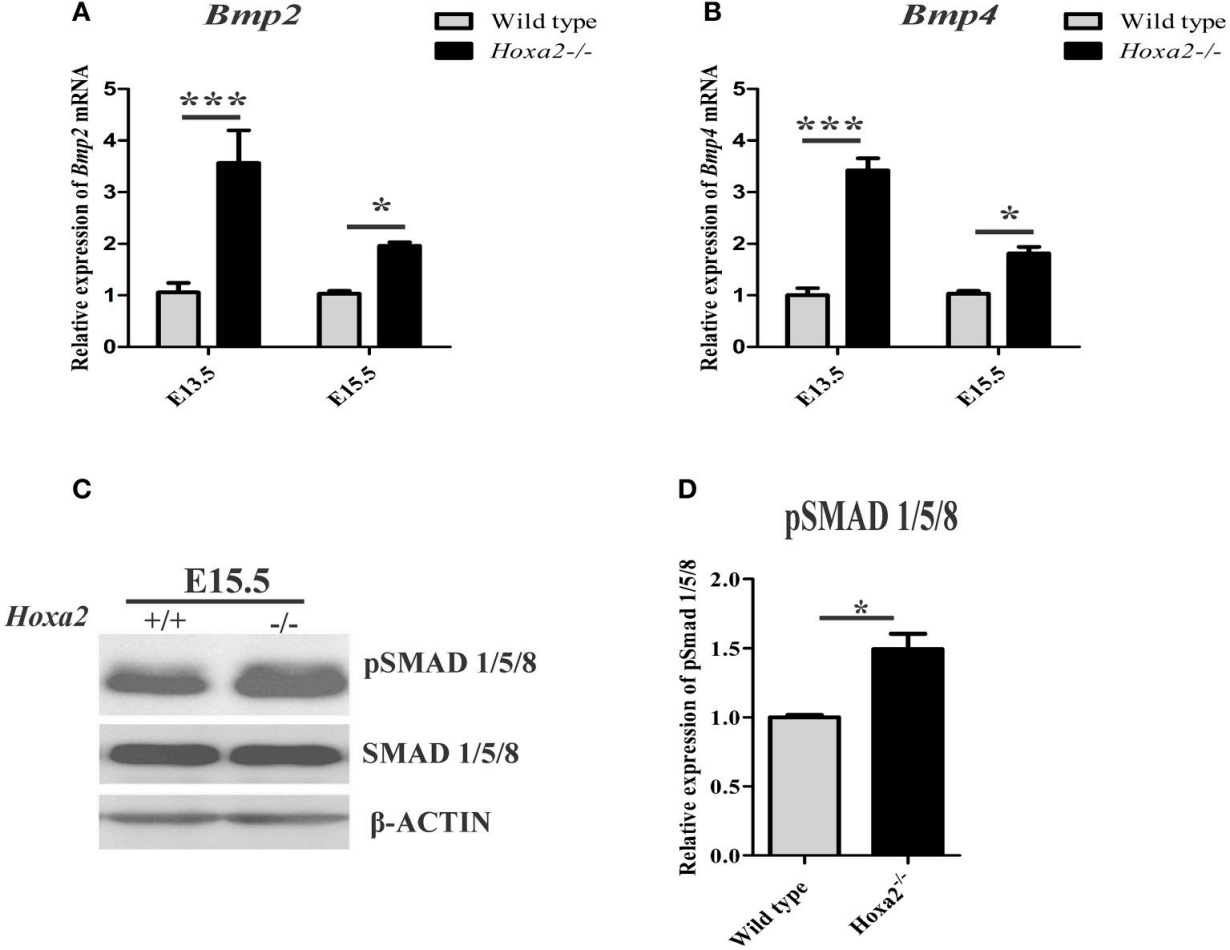
*Hoxa2* regulates canonical BMP signaling in the developing palate. Gene expression of BMP ligands, *Bmp2* **(A)** and *Bmp4* **(B)** were upregulated in *Hoxa2*^−/−^ palatal shelves at E13.5 and E15.5. qRT-PCR data (*n* = 4 biological replicates) were normalized to β*-actin* (mean ± S.E.M; unpaired *t*-test, ^*^p < 0.05; ^***^p < 0.001). Representative immunoblot **(C)** of pSMAD 1/5/8 from the developing palate of wild-type and *Hoxa2*^−/−^ embryos at E15.5. **(D)** Densitometric analysis represents the relative expression of pSMAD 1/5/8 normalized to SMAD 1/5/8 and represented relative to wild-type (*n* = 4 biological replicates; mean ± S.E.M; unpaired *t*-test, ^*^p < 0.05).

To understand the molecular signaling pathways underlying the aberrant cell proliferation and osteogenic differentiation in the *Hoxa2*^−/−^ palatal shelves, BMP signaling was investigated as it is critical for cell proliferation (Zhang et al., 2002) and expression of osteogenic markers in the developing palate (Baek et al., 2011). First, the mRNA expression of BMP ligands critical for osteoblast differentiation such as *Bmp2* and *Bmp4* in the developing palatal shelves was examined. *Bmp2* expression was upregulated to ~3.57-fold at E13.5 and ~1.96-fold at E15.5 in *Hoxa2*^−/−^ palatal shelves compared to wild-type (Figure 5A). Similarly, *Bmp4* expression was upregulated to ~3.42-fold at E13.5 and to ~1.81-fold at E15.5 (Figure 5B). Immunoblotting analyses revealed that canonical BMP signaling mediated by pSMAD 1/5/8 was also upregulated to ~1.5-fold in the *Hoxa2*^−/−^ palate at E15.5 (Figures 5C,D). These results indicate that canonical BMP signaling pathway may be downstream of the *Hoxa2* gene network in palate development.

### Blocking canonical BMP signaling rescues the aberrant cell proliferation and osteogenic differentiation in *Hoxa2^−/−^* MEPM cells

**Figure 6:**
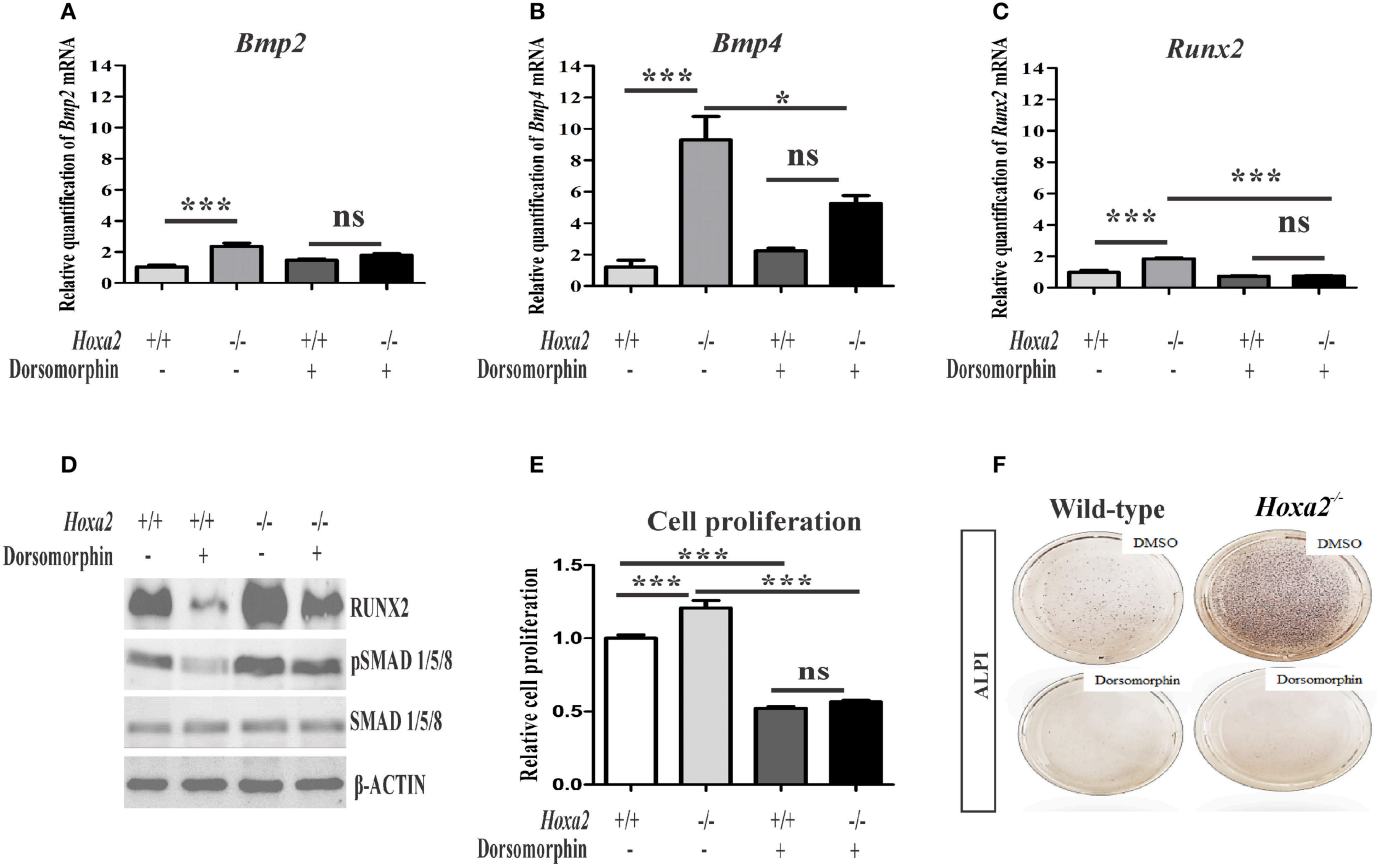
Blocking canonical BMP signaling with dorsomorphin rescues the aberrant osteoprogenitor cell proliferation and osteogenic differentiation in the *Hoxa2*^−/−^ MEPM cells. Dorsomorphin treatment restored gene expressions of *Bmp2* **(A)**, *Bmp4* **(B)**, and *Runx2* **(C)** in the *Hoxa2*^−/−^ MEPM cells close to wild-type levels during osteogenic differentiation *in vitro* at d8. Data represented relative to wild-type MEPM cells treated with DMSO (*n* = 4 biological replicates; mean ± S.E.M; one-way ANOVA followed by Bonferroni *post-hoc* test, ^*^p < 0.05; ^***^p < 0.001; ns, not significant). **(D)** Representative immunoblots showing restoration of RUNX2 and pSMAD 1/5/8 in *Hoxa2*^−/−^ MEPM cells treated with dorsomorphin during osteogenic differentiation *in vitro* at d8 (*n* = 3 biological replicates). **(E)** Cell proliferation analysis in the wild-type and *Hoxa2*^−/−^ MEPM cells treated with DMSO or dorsomorphin during osteogenic differentiation at d3 (*n* = 5 biological replicates; mean ± S.E.M; one-way ANOVA followed by Bonferroni *post-hoc* test, ^***^p < 0.001; ns, not significant). **(F)** ALPI staining revealed that treatment with dorsomorphin nullified the aberrant osteogenic differentiation in *Hoxa2*^−/−^ MEPM cells *in vitro* at d8.

To determine if the upregulated canonical BMP signaling is functionally responsible for the increased mesenchymal cell proliferation and osteogenic differentiation observed in the *Hoxa2*^−/−^ palate, dorsomorphin was used to inhibit BMP signaling during osteogenic differentiation of MEPM cells *in vitro*. Although at higher doses dorsomorphin (10–20 μM) inhibits AMPK signaling (Zhou et al., 2001) and mTOR signaling (Vucicevic et al., 2011), it selectively inhibits BMP signaling at lower doses (Yu et al., 2008). Upon 5 μM dorsomorphin treatment, upregulated mRNA expressions of *Bmp2* (Figure 6A), *Bmp4* (Figure 6B) and *Runx2* (Figure 6C) in the *Hoxa2*^−/−^ MEPM cells were restored to the wild-type levels. Moreover, increased protein expression of RUNX2 and pSMAD 1/5/8 (Figure 6D) in the *Hoxa2*^−/−^ MEPM cells were reduced after dorsomorphin treatment. The increased cell proliferation (Figure 6E) and osteogenic differentiation (Figure 6F) in the *Hoxa2*^−/−^ MEPM cells were also reduced after dorsomorphin treatment. These results indicate that the upregulated canonical BMP signaling is functionally responsible for the increased cell proliferation and osteogenic differentiation during palate development in *Hoxa2*^−/−^ embryos. Altogether, the findings reveal that *Hoxa2* inhibits osteoprogenitor proliferation and commitment, via BMP signaling, to control the spatial and temporal expression of osteoblast markers for proper palatogenesis.

## Discussion

Mice lacking *Hoxa2* exhibit cleft palate (Gendron-Maguire et al., 1993; Rijli et al., 1993; Barrow and Capecchi, 1999) and microtia (Minoux et al., 2013), which are consistent with *Hoxa2* mutations in humans (Alasti et al., 2008). We have previously shown that *Hoxa2* is expressed in the palatal shelves during development (Nazarali et al., 2000) reaching a maximal expression at E13.5 and regulates cell proliferation in the developing palate (Smith et al., 2009). There are several lines of evidence that *Hoxa2* regulates palate development intrinsically (Smith et al., 2009), yet the mechanism is largely unknown. In this study, we have found that *Hoxa2* inhibits BMP signaling dependent osteogenic differentiation spatially and temporally to regulate palate formation. The present study deepens the current understanding of the role of *Hoxa2* in palate formation and the mechanisms underlying the cleft palate phenotype in *Hoxa2*^−/−^ mice linking *Hoxa2*, BMP signaling and osteogenesis.

Our findings here reveal that *Hoxa2* controls the temporal and spatial expression pattern of osteoblast markers in the developing palatal mesenchyme. Ossifying domains characterized by RUNX2 and ALPI were increased in the palatal process of the maxilla and in the palatal process of the palatine bone in *Hoxa2*^−/−^ mice. In contrast, SP7 a marker of mature osteoblasts was expanded only in the palatal process of the maxilla and not in the palatal process of the palatine bone at E16.5. This suggests that cells toward the oral side of the palatal process of the palatine bone are at immature osteoblast stage and may not have developed bone matrix by E16.5. Patterning of the palatal process of the palatine bone and of the maxilla are through independent skeletogenic processes (Baek et al., 2011). The palatal process of the palatine bone ossifies at E13.5, whereas the ossification of the palatal process of the maxilla begins only at E15.5. Consistent with this, qRT-PCR and immunoblot analyses revealed a corresponding upregulation of osteogenic markers in the *Hoxa2*^−/−^ palate at these two critical stages E13.5 and E15.5. In addition, primary *Hoxa2*^−/−^ MEPM cells displayed an increase in osteogenic differentiation and a stage-specific increase in the expressions of the osteoblast-specific transcripts indicating that *Hoxa2* regulates temporal differentiation of mesenchyme cells to osteoblasts in the palate. Together, our results reveal that *Hoxa2* functions as an inhibitor of osteogenic differentiation in the palatal mesenchyme during development. Our findings are in agreement with previous studies showing the role of *Hoxa2* as an inhibitor of bone formation in other craniofacial regions (Kanzler et al., 1998; Dobreva et al., 2006).

Very little is known about the signaling network downstream of *Hoxa2* during palatogenesis. Here, we have demonstrated that *Hoxa2*^−/−^ palatal shelves exhibit upregulated canonical BMP signaling mediated by pSMAD 1/5/8. In addition, the expression of BMP ligands such as *Bmp2* and *Bmp4* are upregulated in *Hoxa2*^−/−^ palatal shelves *in vivo* and in *Hoxa2*^−/−^ MEPM cells *in vitro*. BMP signaling plays a critical role in proliferation (Zhang et al., 2002; Baek et al., 2011) and osteogenic differentiation of the palatal mesenchyme (Wu et al., 2008; Han et al., 2009; Baek et al., 2011). Importantly, abnormal BMP signaling in the palatal mesenchyme leads to cleft palate manifestation (Zhang et al., 2002; He et al., 2008). Inactivation of *Bmpr1a* in the palatal mesenchyme (*Osr2-IresCre; Bmpr1a*^*f*/*f*^) results in submucous cleft palate, absence in the patterning of the palatal process of the maxilla and defective palatal process of the palatine bone (Baek et al., 2011). Genome-wide mapping revealed that *Bmp2, Bmp4* and *Bmpr1a* are possible targets of *Hoxa2* (Donaldson et al., 2012) and HOXA2 protein binds to the intronic region of *Bmp4* (Minoux et al., 2013) in the developing pharyngeal arch2. In this study, dorsomorphin was used to inhibit BMP signaling in the wild-type and *Hoxa2*^−/−^ primary palatal mesenchymal cells during osteogenic differentiation. Dorsomorphin selectively inhibits BMP signaling at lower doses (Yu et al., 2008) and at higher doses dorsomorphin (10–20 μM) also inhibits AMPK signaling (Zhou et al., 2001) and mTOR signaling (Vucicevic et al., 2011). In our study, dorsomorphin treatment not only rescued the upregulated gene expression of osteogenic factors such as *Bmp2, Bmp4*, and *Runx2* but also the aberrant cell proliferation and osteogenic differentiation in the *Hoxa2*^−/−^ MEPM cells. These experiments highlight the involvement of BMP signaling in the abnormal osteoprogenitor cell proliferation and osteogenic differentiation in the *Hoxa2*^−/−^ palate, which could attribute to the cleft palate phenotype in these mutants.

**Figure 7:**
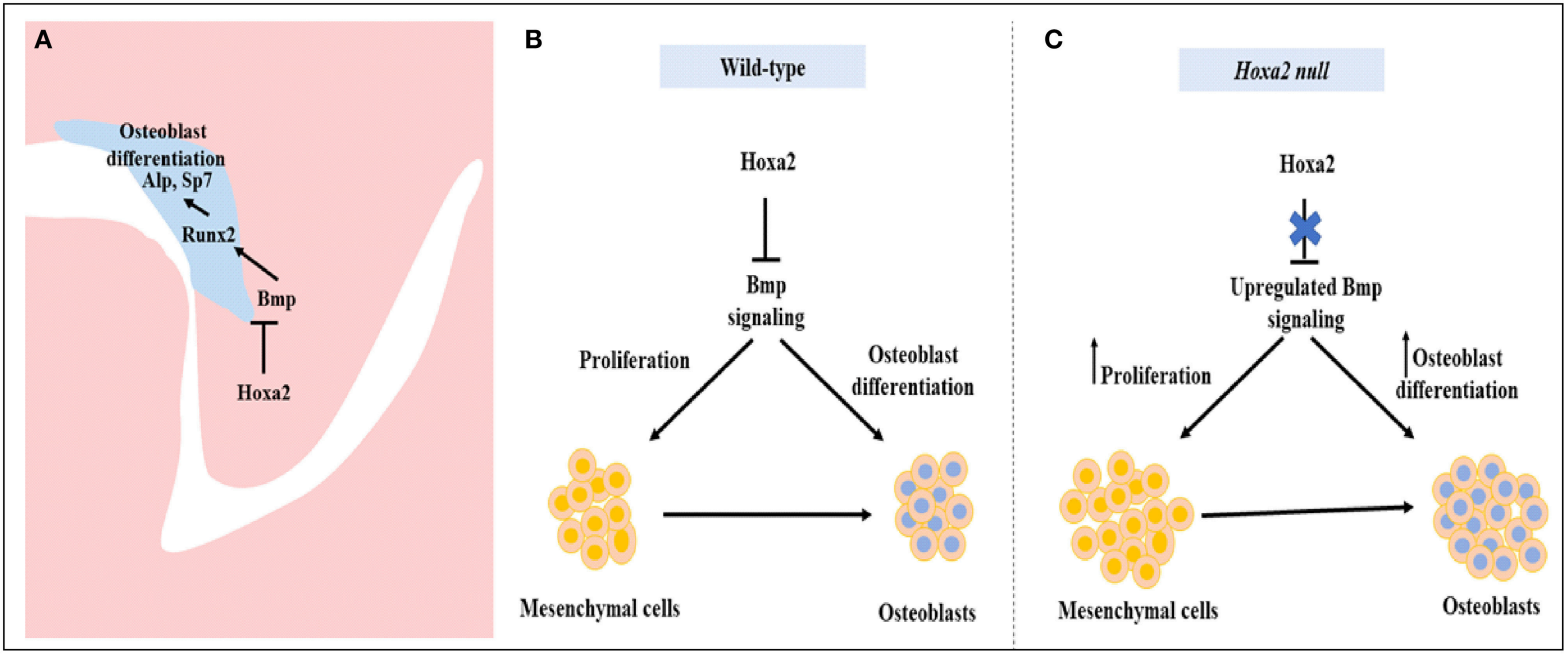
Schematic diagram depicting the role of *Hoxa2* in proliferation and osteogenic differentiation of the palatal mesenchyme. **(A)** *Hoxa2* inhibits canonical BMP signaling in the developing palate, which in turn restricts the expression domain of osteogenic markers such as *Runx2, AlpI*, and *Sp7*. **(B)** In wild-type, *Hoxa2* expression peaks during early palatogenesis to control cell proliferation and to maintain mesenchymal cells in an undifferentiated stage by regulating BMP signaling pathway. **(C)** Loss of *Hoxa2* leads to upregulation of BMP signaling resulting in increased osteoprogenitor cell proliferation and osteogenic differentiation, possibly accounting for the failure in the elevation of palatal shelves resulting in manifestation of cleft palate.

To our knowledge, there is no report available on the characterization of osteoprogenitor cell proliferation and commitment in the palatal mesenchyme during development. In this study, we have unraveled the role of *Hoxa2* in maintaining the palatal mesenchymal cells in an undifferentiated stage by inhibiting osteoprogenitor proliferation and commitment preventing abnormal ossification in the developing palate. Palatal mesenchymal cells derived from CNCC undergo osteogenic proliferation and commit to form osteoblasts (Iwata et al., 2010). Double immunolabeling analyses of RUNX2 and Ki67 at E13.5 revealed that among the total population of mesenchyme cells, there was a significantly higher number of (i) proliferating cells (Ki67-positive cells), (ii) osteoprogenitor cells (RUNX2-positive cells), and (iii) proliferating osteoprogenitor cells (RUNX2-positive /Ki67-positive cells) in the nasal side of the *Hoxa2*^−/−^ palatal shelves compared to the wild-type. In the palatal mesenchyme, increased or decreased cell proliferation could result in failure of the palatal shelves to elevate and reorient above the tongue leading to cleft palate (Bush and Jiang, 2012; Smith et al., 2013). Recent studies show evidence for abnormal osteogenic signaling prior to the elevation of palatal shelves in several well-studied cleft palate mutant mice models including *Pax9*^−/−^ mice (Jia et al., 2017a,b) and *Osr2*^−/−^ mice (Fu et al., 2017). Consistent with our findings here in the *Hoxa2*^−/−^ mice, *Osr2*^−/−^ exhibit increased osteogenic centers of the palatal process of the palatine bone prior to the elevation of the palatal shelves at E13.5 and in addition to defective cell proliferation, enhanced osteogenesis could contribute to cleft palate phenotype in *Osr2*^−/−^ mice (Fu et al., 2017). In addition, RNA-Seq data from *Osr2*^−/−^ palatal shelves revealed upregulation of several positive regulators of osteogenesis including *Runx2, Runx3, Sp7*, and Bmp ligands- *Bmp3, Bmp5*, and *Bmp7*. Furthermore, *Pax9*^−/−^ mice exhibit reduced cell proliferation and osteogenesis in the developing palate (Jia et al., 2017a). Restoration of reduced cell proliferation and osteogenesis by Wnt agonists (Dkk inhibitors) rescued the cleft palate phenotype in *Pax9*^−/−^ mice (Jia et al., 2017a). The increase in cell proliferation in the nasal side of the *Hoxa2*^−/−^palate indicates a strong role for *Hoxa2* in the spatial maintenance of mesenchymal cells in an undifferentiated state for temporal coordination of osteoblast differentiation (Figure 7). Our findings here exemplify the regional heterogeneity in proliferation and osteogenic differentiation by *Hoxa2* along the oral-nasal axis in the palatal mesenchyme prior to the elevation of palatal shelves. Our data argue that improper BMP signaling leading to the increased osteoprogenitor cell proliferation and commitment could be a reason for the cleft palate pathogenesis in the *Hoxa2*^−/−^ mice. Further studies are needed to address if the cleft palate phenotype in the *Hoxa2*^−/−^ mice could be rescued using other mutant mice with impaired osteogenesis.

Our data demonstrate that *Hoxa2* inhibits osteoprogenitor cell proliferation and osteogenic commitment via modulating BMP signaling in the mouse embryonic palatal mesenchyme. *Hoxa2* regulates spatial and temporal programs of osteogenesis by maintaining mesenchymal cells in an undifferentiated stage until osteogenic clues arrive. In conclusion, our findings provide new insights into the signaling mechanism underlying the role of *Hoxa2* during embryonic palate development.

## Author contributions

AN conceived and coordinated the study. PI designed the study, performed experiments, analyzed data and wrote the manuscript. AN and PI proofed, revised the manuscript for critical content and interpretation of data.

### Conflict of interest statement

The authors declare that the research was conducted in the absence of any commercial or financial relationships that could be construed as a potential conflict of interest.

## Abbreviations

AlpI: alkaline phosphatase I
ARS: Alizarin red S
BMP: bone morphogenetic protein
CNCC: cranial neural crest cells
d: day
E: embryonic day
MEPM: mouse embryonic palatal mesenchyme
pSMAD 1/5/8: phosphorylated SMAD 1/5/8
qRT-PCR: quantitative real-time PCR
Runx2: runt-related transcription factor 2.
